# The Question of Operation in Chronic Non-Suppurative Ear Disease

**Published:** 1897-05-15

**Authors:** 


### THE QUESTION OF OPERATION IN CHRONIC NON-SUPPURATIVE EAR DISEASE.

Few patients are more anxious for operation, in other words, for relief at any price, than those who suffer from chronic non-suppurative ear disease, with its distressing accompaniments of deafness and tinnitus; and it often becomes a matter of no small difficulty to decide whether to advise patience or operative interference.

To understand what amount of improvement may be expected from operation on the middle ear, it is necessary to bear in mind that the whole auditory apparatus of tympanic membrane and ossicles, down to the oval window into the inner ear, although a great assistance to audition is not essential to it; so that even if operation should go to the extent of removing the whole of these accessories, moderate hearing may be preserved; although, of course, the protection afforded by the membrane will be lost, and various faculties of accommodation will be abolished.

As to the broad question whether such operations are worth while, the *Practitioner*, after making inquiries from leading aural surgeons in all parts of the world, with the result of eliciting a wide variety of opinions, says, "It cannot be denied that with a few exceptions aural surgeons either are averse from all operative interference, or at present prefer to keep an open mind on the subject, with perhaps a leaning to one operation or another in suitable cases."

There is, however, no doubt that some cases are improved, more especially in regard to their tinnitus, by operation. The uncertainty is such, however, that no one should undergo such operations without thoroughly understanding that he may possibly be made worse by the proceeding. There are many to whom this will be no deterrent, for some patients suffering from tinnitus not only would undergo anything on the chance of relief, but entirely disbelieve that they could possibly be made worse than they are, so oppressed do they feel by the constant presence of their malady.

It is the presence of unbearable tinnitus, far more than the existence of deafness, which drives people to ask for operation. Unfortunately, the relief of tinnitus by such measures is quite as problematical as the relief of deafness. Still, it always may occur, the division of some slight adhesion liberating the point where pressure has arisen.

As to the form of operation which may be required, we may say at once that, if real benefit is to be aimed at, the patient must usually be prepared to submit to something much more radical than the making of a mere opening in the membrane. An exploratory myringotomy, however, is of great service in indicating whereabouts the lesion which is doing the mischief lies.

Roughly one may say that where the sonorous vibrations are interrupted by thickening and rigidity in the membrane and external ossicles there is hope that the removal of the membrane and of the two larger bones, as advocated by Dr. Sexton of New York, may give to the patient that modicum of hearing of which so mutilated an organ is capable; while where tinnitus is the difficulty, and is set up by disease in that position, either that operation or separation of the stapes from the incus, and thus from the membrane, may possibly relieve the condition.

Where, however, the stapes itself is affected, and where the oval window is interfered with, none of these proceedings will remedy the disease. In such cases nothing short of loosening the rigidity affecting this opening is likely to do good. Mobilisation or removal of the stapes has now been done in many cases, and in a few with good results; perhaps with better result to the tinnitus, as a rule, than to the deafness.

It has to be borne in mind, however, that the condition which leads to the sclerotic changes which are found in these cases of chronic middle-ear disease is a progressive one, and that the tendencies of the malady will not always be checked by merely setting free the adhesions produced by it. On the whole, we may anticipate a better final result in those cases in which the symptoms are relieved by removal of the membrane, and free opening up of the whole of the tympanic cavity, than by operations which involve a less permanent and complete interference with the outer parts.

